# A rare case of dual expression of CD5 and CD10 in primary central nervous system diffuse large B‐cell lymphoma with prominent starry sky appearance

**DOI:** 10.1002/ccr3.3293

**Published:** 2020-09-15

**Authors:** Colin McCorkle, Vincent Graffeo

**Affiliations:** ^1^ Marshall University Joan C. Edwards School of Medicine Huntington West Virginia USA

**Keywords:** CD10, CD5, central nervous system lymphoma, diffuse large B‐cell lymphoma, starry sky

## Abstract

This case highlights the need for both tissue biopsy for diagnosis in suspected CNS malignancy and comprehensive immune profiling for accurate subclassification.

## Graphical abstract

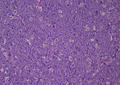

## 1 INTRODUCTION

A 69‐year‐old woman presented with a several‐month history of progressively worsening aphasia and new‐onset seizure. A computed tomography (CT) scan revealed a hypodense left temporal lobe mass with surrounding vasogenic edema; initially, concern was high for a metastatic tumor. A biopsy was performed, and pathology demonstrated a high‐grade diffuse large B‐cell lymphoma with double expression of both CD5 and CD10.

Central nervous system lymphoma (CNS) is a rare disease; primary CNS lymphoma accounts for 2 to 3% of all brain tumors. Median patient age for primary CNS lymphoma is 56 years with a slight male predominance.^1^ Many patients have some evidence of an associated immunodeficiency state. The majority of primary CNS lymphomas are diffuse large B‐cell lymphoma, but T‐cell lymphomas are also known to occur in the CNS. We report a case of a primary CNS diffuse large B‐cell lymphoma with a rare combination of co‐expression of both CD5 and CD10 on the malignant cells.

## 2 CASE PRESENTATION

**FIGURE 1:**
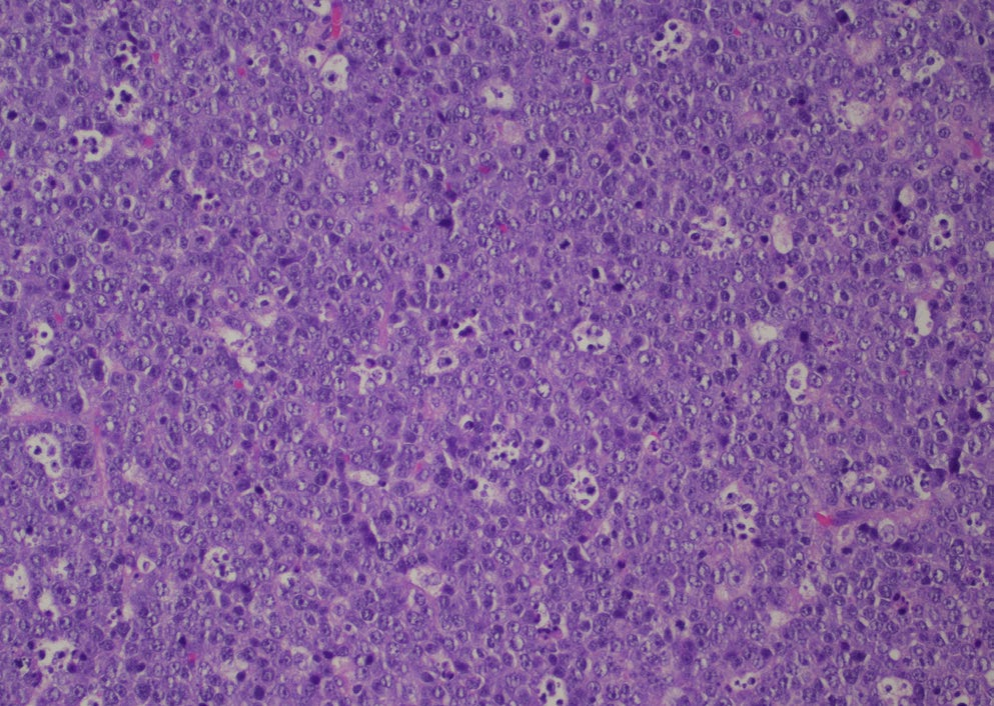
Hematoxylin and eosin stain shows a diffuse proliferation of larger malignant lymphocytes with a starry sky pattern

**FIGURE 2:**
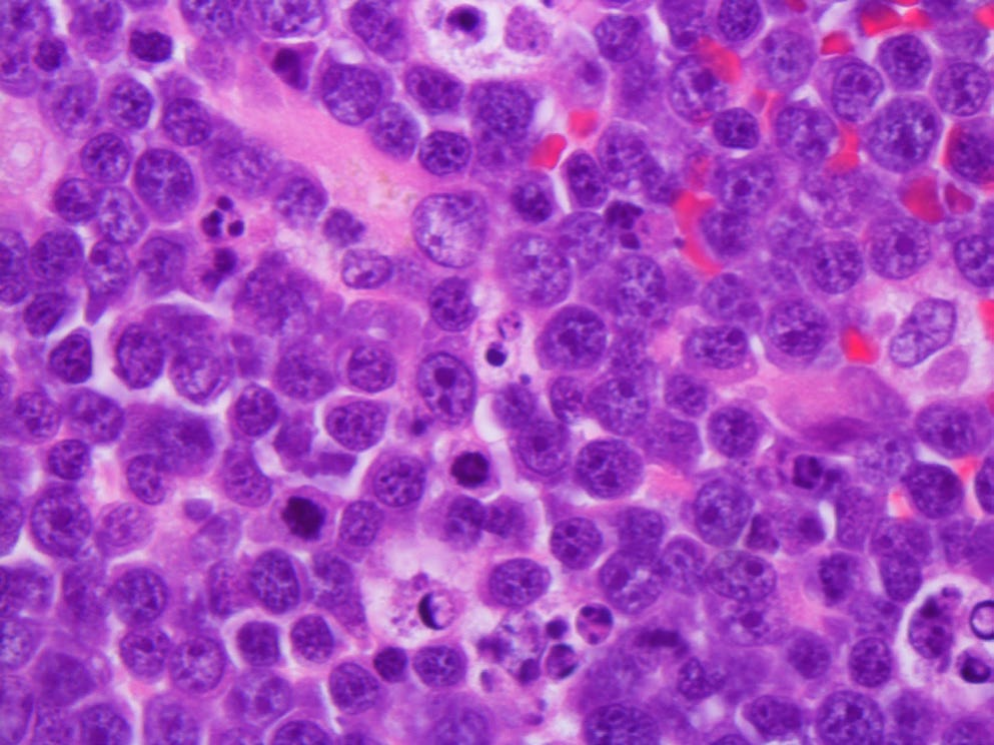
High magnification of malignant lymphocytes

**FIGURE 3:**
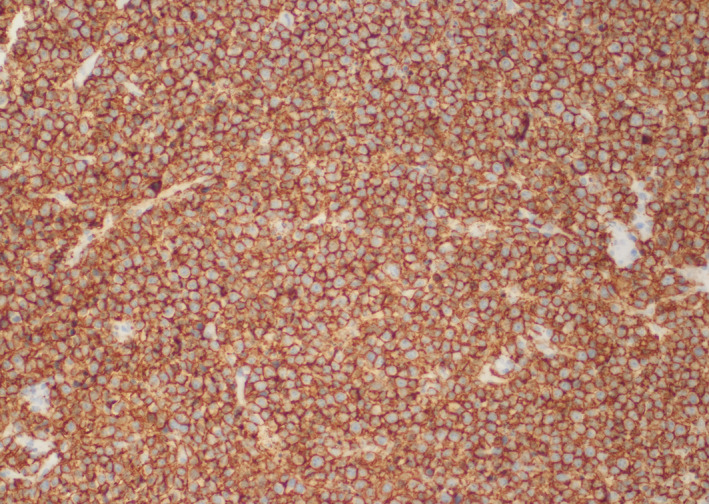
CD20 immunostain highlights malignant B lymphocytes in a strong and diffuse manner

**FIGURE 4:**
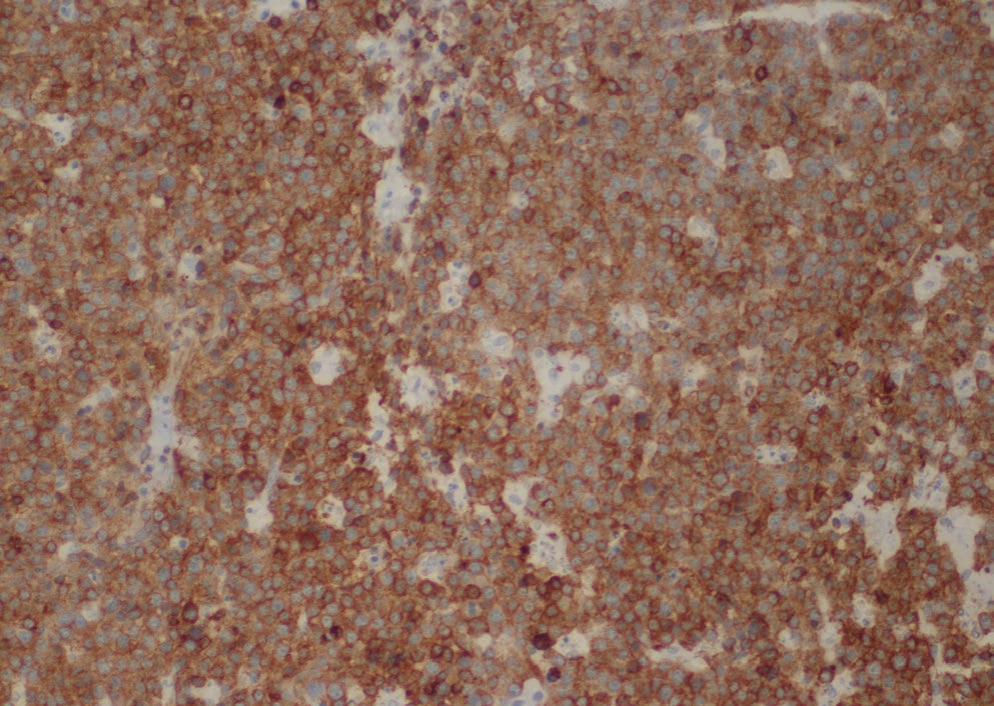
CD5 immunostain shows diffuse positive staining in the malignant B cells

**FIGURE 5:**
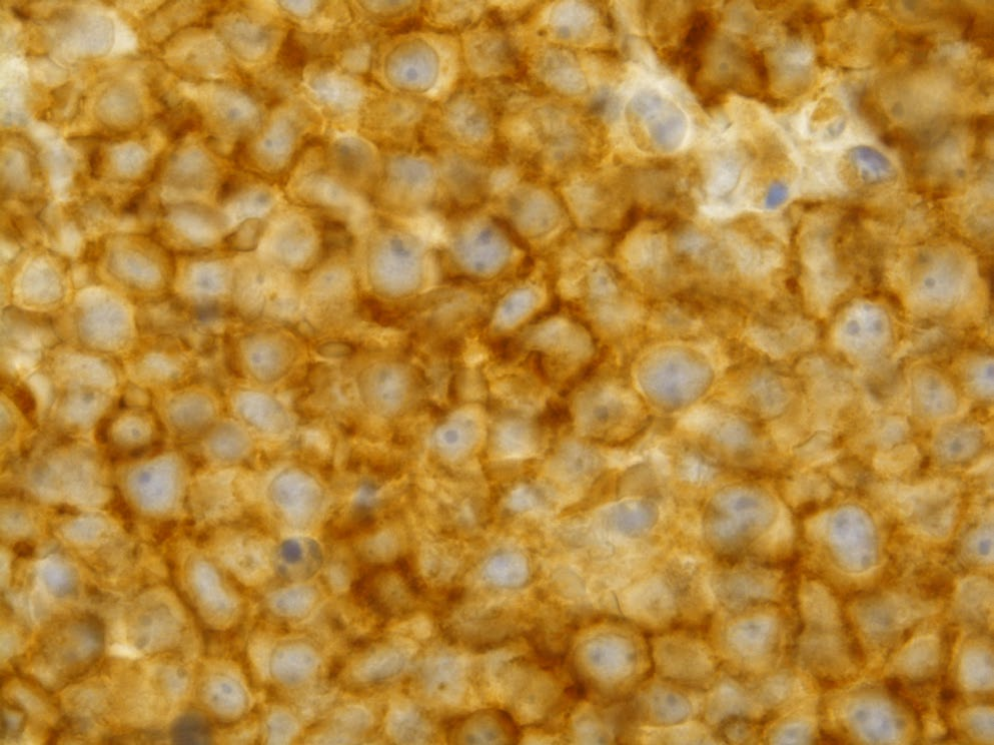
CD10 immunostain is also diffusely positive in malignant B cells that co‐express CD5

The patient is a 68‐year‐old woman with a history of hypothyroidism and vertigo presented with an onset of seizures. A few months prior to presentation, the patient's family had noticed she was having progressive deterioration of balance and the development of ataxia, weakness, and expressive aphasia. She denied any weight loss or constitutional signs. There was no known history of immunodeficiency or medications that would impair immunocompetence. Physical examination was remarkable for a positive Babinski sign, bilateral lower extremity weakness, and difficulty with speech. Complete blood count (CBC) was normal, and CT and magnetic resonance imaging (MRI) were performed. MRI showed a 1.4‐cm. mass in the left temporal lobe with surrounding vasogenic edema raising concern for possible metastatic neoplasm. Chest abdomen and pelvis CT and MRI and positron emission tomography (PET) were performed as a part of a complete evaluation for a primary non‐CNS malignancy. There was no evidence of a primary lesion to confirm the impression of metastatic disease. Because of the location of the lesion, a biopsy was not initially indicated and she received 2 doses of CyberKnife radiosurgery. However, the tumor did not respond to treatment and repeat MRI showed evidence of a new lesion in the left frontal region. Neurosurgery was consulted and subsequently performed a left temporal craniotomy with resection of the left temporal and left frontal masses. The histopathology showed a lymphoid malignancy with large pleomorphic cells, marked nuclear enlargement, prominent nucleoli, and prominent starry sky appearance (Figures 1 and 2.) Immunohistochemistry showed that the tumor cells were positive for CD20, CD5, CD10, and MUM‐1. CD3 was negative in the tumor (Figures 3, 4 and 5) cells. Additionally, the tumor cells were positive for BCL‐2 and BCL‐6. A semiquantitative Ki‐67 immunostain was performed and labeled nearly 100% of tumor cell nuclei. BCL‐1/Cyclin D‐1 immunostain was negative. FISH studies were negative for MYC rearrangement, negative for a rearrangement of BCL‐2, and negative for a CCND1‐IgH translocation. To evaluate for an immunodeficiency‐associated lymphoproliferative disorder, we performed in situ hybridization for Epstein‐Barr virus (EBV). The EBV result was negative. Appropriate treatment for a high‐grade CNS lymphoma was initiated, but the patient expired 15 days after admission.

## 3 DISCUSSION

Mature B‐cell neoplasms usually express CD5 or CD10 or neither in addition to expressing a lineage defining marker such as CD19 or CD20. Correct assignment of a diagnostic category to lymphoid malignancy usually involves determination of the immunophenotype of the malignant cells by either one of two diagnostic modalities, immunohistochemistry, or flow cytometry.^2^ The majority of B‐cell lymphomas (>80%) will be either CD5‐ or CD10‐positive, and in concurrence with histopathologic diagnosis, molecular, and cytogenetic features, the subtype of lymphoma can be further specified within the currently accepted World Health Organization Classification of Tumors.^3^ Specific and accurate lymphoma subtype diagnosis is vitally important for both prognostication and treatment purposes.^3^ CD5‐positive B‐cell neoplasms are most commonly seen in chronic lymphocytic leukemia/small lymphocytic lymphomas (CLL/SLL) and mantle cell lymphoma (MCL). Rare cases of CD5‐positive marginal zone lymphomas have been reported. Occasionally, CD5 is expressed on de novo diffuse large B‐cell lymphoma. CD10 expression is generally identified on lymphoblastic lymphoma (LBL), follicular lymphoma (FL), Burkitt lymphoma, and a subset of diffuse large B‐cell lymphoma (LBCL). However, there are exceptions such as CD5‐ MCLs,^5^, ^6^, ^7^, ^8^ CD10‐ FLs,^5^, ^6^, ^7^, ^8^ and LBCLs that are CD5‐ or CD10‐positive.^2^, ^9^ These exceptions to the more usual and expected patterns of antigen expression can lead to misdiagnosis and mismanagement of the associated malignancy. Further evaluation including extensive immunophenotyping and genetic/fluorescence in situ hybridization (FISH) to evaluate for diagnostic translocations involving genes such as IgH, CCND1, BCL‐2, BCL‐6, and MYC may be necessary to overcome uncertain classification based on limited morphologic and immunophenotypic data..^2^

Simultaneous CD5+ and CD10+ neoplasms are extremely rare and are estimated to make up only 0.4% of all B‐cell lymphomas.^2^ The co‐expression of CD5 and CD10 cell surface markers is highly unusual as these antigens are never expressed concurrently in the natural development of a B cell. Due to the rarity of CD5+ CD10+ lymphomas, there is not a predictable clinical course or outcome described in the previous literature. The morphology previously described of these neoplasms by Dong et al^4^ has a wide range of histopathology ranging from pseudofollicles to a highly mitotic state with a high Ki‐67 expression and starry sky appearance resembling Burkitt lymphoma. We present a rare case of a diffuse large B‐cell lymphoma expressing both CD5 and CD10 with a marked starry sky appearance. Given the morphology in this case, evaluation of the MYC oncogene status was necessary. Notably, this lymphoma was negative for a t(8;14) translocation or MYC rearrangement which, if identified, would support a Burkitt or Burkitt‐like lymphoma. This case highlights the need for both tissue biopsy for diagnosis in suspected CNS malignancy and comprehensive immune profiling for accurate subclassification.

## CONFLICT OF INTEREST

None declared.

## AUTHOR CONTRIBUTIONS

All authors approved the final manuscript as submitted and agreed to be accountable for all aspects of the work.

## ETHICAL APPROVAL

This case report used only de‐identified archival case material; no additional institutional approval was required.
